# Effect of Material Composition and Alkali Activator Modulus on Mechanical Properties and Reaction Process of Coal-Based Solid Waste Geopolymer

**DOI:** 10.3390/ma19204369

**Published:** 2026-10-17

**Authors:** Ling Luo, Kaijun Chang, Yongjun Qin, Zhenji Long, Jie Ran

**Affiliations:** 1 School of Civil Engineering and Architecture, Xinjiang University, Urumqi 830047, China; 2 Xinjiang Civil Engineering Technology Research Center, Xinjiang University, Urumqi 830047, China

**Keywords:** geopolymer, coal-based solid waste, alkali-activation kinetics, microstructure

## Abstract

This study investigates the effects of coal-based solid waste/ground granulated blast furnace slag mass ratios (90/10,70/30,50/50) and water glass modulus (1.0, 1.2, 1.5) on geopolymer fluidity, setting time, and compressive strength. Fly ash (FA), coal gangue (CGP; 20% of coal-derived solid waste), and finely ground granulated blast furnace slag (GGBS) particles are used as composite precursors. The reaction mechanism was elucidated through XRD, TGA, and SEM-EDS analyses. Slag content is the main influencing factor, as increasing its proportion from 10% to 50% enhances the 28-day compressive strength of the geopolymer by 250%. Increased slag promotes the formation of additional C-A-S-H gel and results in a denser matrix. In contrast, excessive amounts of coal gangue can lead to an increase in porosity due to its high crystallinity and low reactivity, which inhibits improvements in mechanical properties. The optimal formulation (coal-based solid waste/ground granulated blast furnace slag 50/50; activator modulus 1.5) achieves a 28-day compressive strength of 32.6 MPa. This study provides theoretical and technical support for the rational utilization of coal-based solid waste and the development of cementitious materials from industrial solid waste.

## 1. Introduction

Against the backdrop of global efforts to achieve carbon neutrality and promote green development models, the low-carbon transition in the building materials industry has become a key development direction [1,2]. Conventional Portland cement, the world’s most extensively utilized cementitious material, is associated with significant energy consumption and carbon emissions during its manufacturing process [3]. Each ton of cement produced emits approximately 0.8 tons of CO_2_, accounting for 8% to 10% of total annual carbon emissions. Consequently, the development of eco-friendly alternative materials has emerged as a research hotspot in both academia and industry.

Geopolymer [4], a novel inorganic cementitious material, has gradually become a research focus since its introduction by French scientist Joseph Davidovits due to its unique green attributes and outstanding properties. The core mechanism involves chemical reactions between alkaline activators (e.g., sodium hydroxide or water glass) and silicate precursors (e.g., fly ash (FA) [5], GGBS [6], coal gangue (CGP) [7], etc.), forming a three-dimensional Si–O–T gel structure. This architecture not only matches the mechanical properties of cement but also exhibits enhanced corrosion resistance, high-temperature stability, and volume stability.

Fly ash is commonly categorized based on its calcium content into high-calcium and low-calcium types. Kucukgoncu [8] found that the proportion of alkali activator to binder significantly governs the behavior of low-calcium fly ash-based geopolymer concrete. Subjected to 800 °C, specimens experienced mass reduction, a sharp drop in compressive strength, and matrix porosity, indicating severe damage to mechanical properties under high temperatures. The aforementioned ratio directly determines the thermal stability of the material. Dineshkumar and Umarani [9] focused on the impact of sodium hydroxide (SH) and sodium silicate (SS) composite alkali activators on FA–GGBS-based polymer: when the SH concentration increased from 2.5 M to 5.0 M and the SS ratio rose from SH:SS = 1:1 to 1:3, the slurry consistency decreased, and setting time shortened. Increasing GGBS content further accelerated setting and hardening, providing a basis for formulation design and construction optimization of such materials. Temuujin and Davaabal [10] argued that the potential activity of low-calcium fly ash is closely related to its active silicon content, glass content, and fineness distribution. In activator studies, water glass, as the most commonly used alkali activator, has been widely validated for its activating effects. Bocullo [11] discovered that the optimal molar ratio of SiO_2_ to Na_2_O ranges from 1.5 to 2.3, under which conditions the 28-day compressive strength of the polymer can reach 42.33–56.43 MPa. Zurinskas et al. [12] investigated the effect of woody biomass fly ash (BFA) on the properties of alkali-activated geopolymer binders. Matrices were prepared using diatomaceous earth and iron dust, with 10–30% BFA incorporated, and characterized using XRD and FTIR. The 10% BFA specimen exhibited the highest compressive strength (53 MPa) but had limited thermal stability; increasing the BFA content improved water resistance but reduced strength. The material properties were synergistically regulated by the gel, phases, and microstructure. Wu [13] explored the influence of sodium hydroxide concentration on alkali-activated FA, demonstrating that, at 14 M sodium hydroxide concentration, a Na_2_SiO_3_/NaOH ratio of 1.5, and a liquid-to-solid ratio of 0.5, a 28-day compressive strength of 21.5 MPa was reached. When the NaOH viscosity was increased to 16 M while all other conditions remained unchanged, the flexural strength reached an optimal value of 5 MPa. Additionally, alkali-activated fly ash mortar displays considerably better durability than ordinary Portland cement (OPC) when exposed to sulfate solutions and marine environments.

Granulated blast furnace slag, as a highly reactive precursor in geopolymer systems, efficiently releases active elements in alkaline environments with rapid reaction rates, producing geopolymers characterized by high strength and rapid setting properties [14]. Kumar et al. [15] demonstrated that slag modification improves the polymerization behavior of fly ash geopolymers, primarily generating A-S-H and C-S-H gels with varying Si/Al and Ca/Si ratios while establishing a correlation mechanism between microstructure and macroscopic performance. Jiang et al. [16] observed that, after 120 days of immersion in 2.5% sodium sulfate solution, the compressive strength of geopolymer samples exhibited an initial increase, followed by a decline, indicating excellent resistance to sulfate erosion. Shi et al. [17,18] revealed that increasing alkali dosage and water glass modulus enhances the carbonation resistance of alkali-activated slag mortar, though water glass modulus significantly affects mortar expansion and pore solution alkalinity: under higher moduli, the expansion rate of alkali-activated slag mortar increases with alkali dosage. Babaee and Castel [19] found that calcium components in precursors significantly reduce chloride ion diffusion rates, while higher alkali concentrations generally increase porosity and chloride ion diffusion coefficients. Fang and Zhang et al. [20] conducted in-depth research on the multiscale micro-cementation structures of alkali-activated FA–GGBS materials, identifying N-A-S-H and C-A-S-H gels as primary reaction products, with minor amounts of N-C-A-S-H gels present. Research indicates that the elasticity of reaction products and their relative volume fraction are key factors determining the macroelasticity of materials. The regulation of macroscopic strength properties primarily depends on two critical factors: porosity and pore size distribution.

Currently, the application of certain geopolymer precursor materials remains limited, particularly in the efficient utilization of inert solid waste such as coal gangue. Due to its strong structural stability and high chemical inertness, raw coal gangue exhibits low reactivity in alkaline environments [21]. Xiao et al. [22] employed coal gangue as fine aggregate in concrete preparation. Results indicated that both workability and tensile strength decreased as the gangue content increased. Gao [23] concluded that coal gangue can be used as coarse aggregate for the production of green concrete, and its physicochemical properties generally meet the requirements for aggregate. A high substitution rate alters water absorption characteristics and reduces mechanical properties, making it unsuitable for underground engineering; its porous structure enhances thermal insulation but compromises durability. The application of this aggregate in structural members is limited, and steel-tube concrete is the preferred form. Long et al. [24] developed high-performance foundation treatment materials using coal gangue, showing that increased coal gangue content significantly enhanced acid resistance in acidic solutions, indicating substantial potential for foundation and subgrade treatment. Cui et al. [25] synergistically modified β-hydroxypropylglycol (β-HPG) with thermally activated coal gangue (CCG) and carbonized slag (CS), finding that a 3:1 CCG/CS ratio increased 28-day dry compressive strength and softening coefficient by 158.51% and 115.63%, respectively, while significantly optimizing pore structure and reducing harmful porosity. Ma et al. [26] demonstrated that Al_2_O_3_ formed through dehydroxylation of kaolinite in coal gangue exhibits strong volcanic ash activity, capable of reacting with chloride ions to form calcium aluminate hydrate. Additionally, Guo et al. [27] found that, when CGP was below 30%, the resulting paste’s compressive strength surpassed 40 MPa. However, Lui [28] conducted systematic research on the mechanical and durability properties of coal gangue-based concrete. They found that compressive strength gradually decreased with increasing coal gangue content. When the admixture content exceeded 50%, the compressive strength of specimens at 90 days showed a decline compared to that at 28 days. Bilek et al. [29] investigated the early nucleation, chemical phase evolution, and system performance of alkali-activated cementitious materials, emphasizing that raw material types, chemical composition, reactivity, and curing conditions are crucial for chemical control of geopolymer systems. Specifically, the rapid dissolution of highly reactive SiO_2_ and Al_2_O_3_ provides high concentrations of silicates and aluminates, which react with Ca^2+^ to form C-A-S-H gels, thereby shortening setting time. This explains why coal gangue–slag geopolymer systems often exhibit excessively rapid setting characteristics.

Current research has focused on property regulation and activation mechanisms of single or composite precursor-based geopolymer systems. However, the systematic coupling of critical parameters—including coal-based solid waste (fly ash + coal gangue) ratios, water glass modulus, and component interactions—on gel formation, structural compactness, and final performance remains unclear. This study employs fly ash, coal gangue, and blast furnace slag as composite precursors. By optimizing the mass ratio of coal-derived solid waste to slag and adjusting the alkali activator modulus, combined with microscopic analysis (XRD, TGA, SEM-EDS), we identify the optimal formulation. Through coupled validation of geopolymerization kinetics and TG thermal analysis, a new perspective was proposed: a “mild and sustained” hydration regime is more conducive to the formation of a dense gel network. Furthermore, through quantitative SEM-EDS analysis, the Ca/Si ratio of the C-A-S-H gel was established for the first time as a key indicator for regulating the microstructure and predicting the macroscopic strength of this system. The results demonstrate that the system achieves both geopolymer strength and workability while revealing the interfacial interactions and reaction kinetics among its alkali-activation kinetics.

## 2. Materials and Methods

### 2.1. Raw Materials Characterization

**Figure 1:**
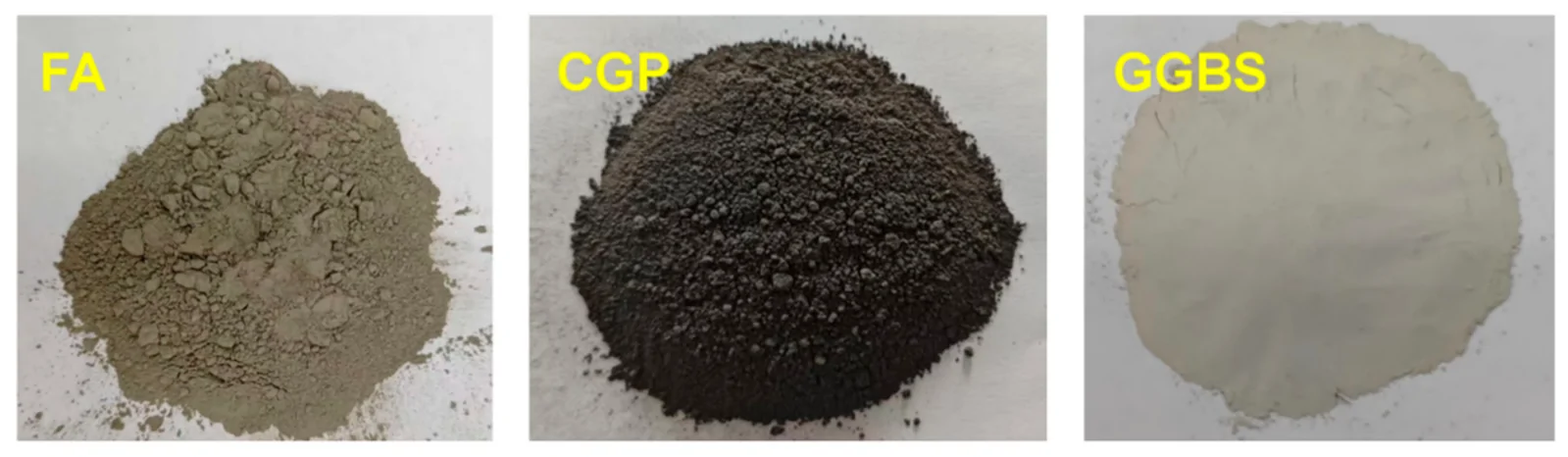
Physical morphology of the cementitious material at room temperature.

This study prepared a geopolymer using CGP, FA, and GGBS as precursors, specifically selecting Class II fly ash. CGP was provided by Xinjiang Mengke Energy Technology Co., Ltd. (Urumqi, Xinjiang, China), while FA and GGBS were supplied by Bayi Iron and Steel Plant in Urumqi, Xinjiang, China. The bulk densities of CGP, FA, and GGBS, determined per standard GB/T 208-2014 [30], were 0.87, 2.34, and 2.68$\;{g/cm}^{3}$, respectively. The chemical compositions are shown in Table 1. The physical morphology of the materials at room temperature is illustrated in Figure 1.

**Figure 2:**
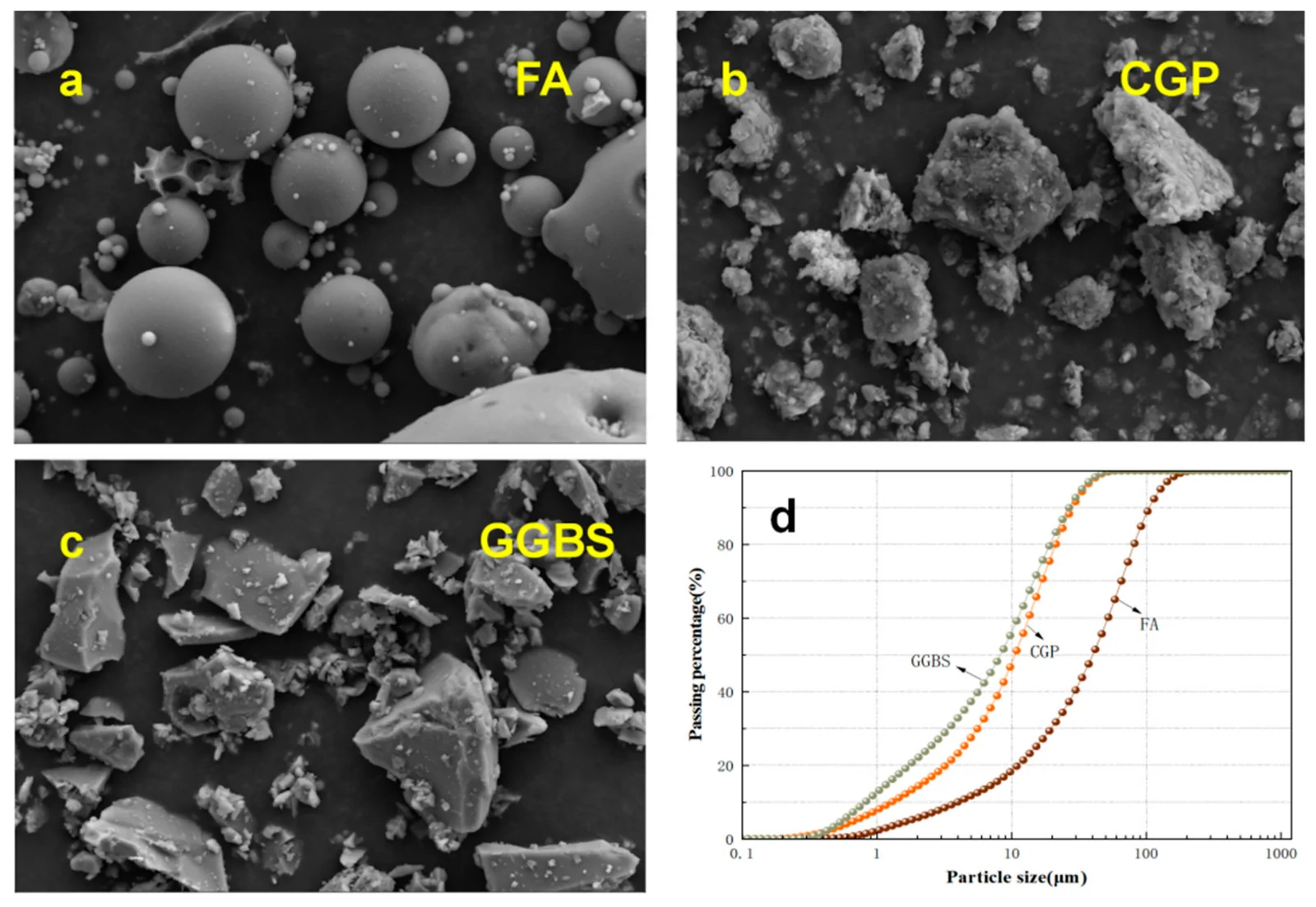
SEM image of the gelling material and its particle size cumulative distribution. e.g., (**a**) Microscopic morphology of FA; (**b**) Microscopic morphology of CGP; (**c**) Microscopic morphology of GGBS (**d**) Cumulative particle size distribution chart.

Particle size distribution (PSD) analysis for CGP, FA, and GGBS was conducted using a Bettersize 3000 Plus laser image particle size analyzer (Dandong Baite Instruments Co., Ltd., Dandong City, Liaoning Province, China). The PSDs of all cementitious materials are shown in Figure 2d, with median particle sizes (D50) of 11.91 µm, 44.99 µm, and 9.31 µm for CGP, FA, and GGBS, respectively. Figure 2 shows SEM images of CGP, FA, and GGBS, respectively. The images reveal that CGP and GGBS exhibit irregular flake-like structures, while FA appears as spherical particles.

This study employed a composite silicate solution to construct an alkaline excitation system, The alkali activator was prepared by mixing sodium hydroxide particles (96% wt.%) and commercial sodium silicate solution in a certain proportion. The chemical composition of sodium silicate was Na_2_O (9.41 wt.%) and SiO_2_ (23.49 wt.%), and the original modulus was 2.58. Due to the solution’s high viscosity, sodium hydroxide was used to adjust its modulus before it could be used in the experiment. After adjustment, the solution had moduli of 1.0, 1.2, and 1.5, respectively, with a concentration of 37%. The preparation procedure was as follows. Initial water glass was mixed with a measured amount of NaOH granules and ultrapure water, and the mixture was stirred continuously for 30 min until the solution became homogeneous. It was then allowed to stand for 24 h at a constant temperature of (25 ± 2) °C. Once the ionic state had stabilized, the solution was used for subsequent experiments; see Table 2 for detailed proportions.

### 2.2. Mix Design

Based on preliminary findings and pilot tests, this study established a mixed design with nine coal-based solid waste/ground granulated blast furnace slag mass ratios and varying alkali activator modulus values. The coal-based solid waste/ground granulated blast furnace slag mixture was configured at three levels (90/10, 70/30, and 50/50), with a fly ash to coal gangue ratio of 4:1 in the coal-based solid waste. The activator modulus Ms was set at 1.0, 1.2, and 1.5, respectively, while maintaining a fixed liquid-to-solid ratio of 0.8. The slurry test specimens were prepared according to the above proportions, and the effects of these factors on flowability, setting time, and compressive strength were analyzed. Detailed settings for the experimental variable are presented in Table 3.

### 2.3. Sample Preparation

**Figure 3:**
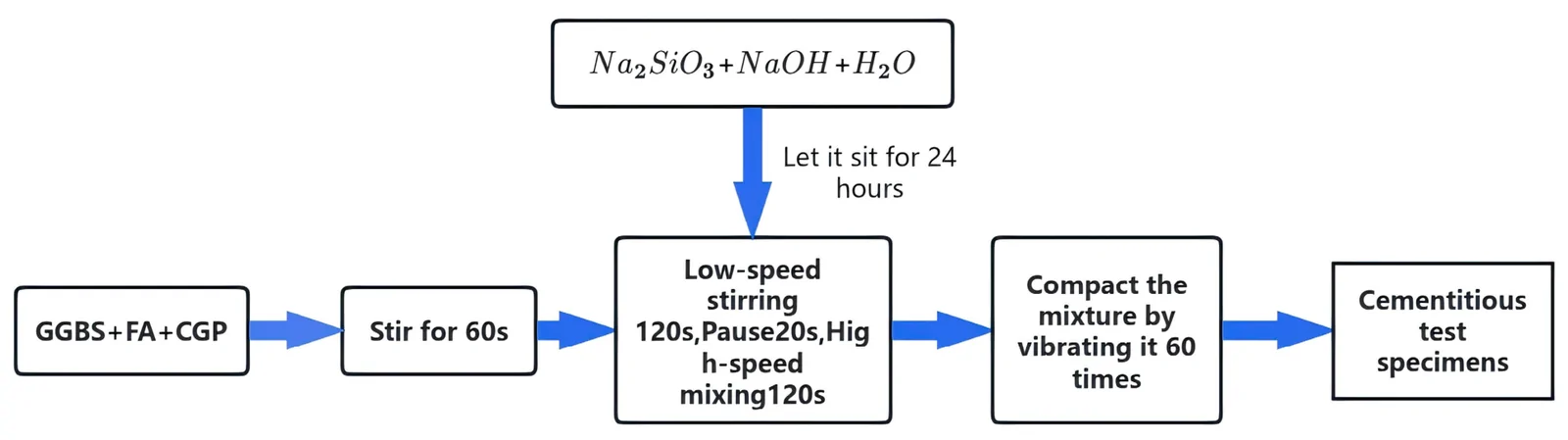
Flowchart of specimen preparation.

Figure 3 shows the schematic diagram of the test procedure. NaOH and water glass were mixed to prepare the activator with the target modulus, which was then cooled and set aside. Coal gangue, fly ash, and blast furnace slag were weighed according to the specified proportions and dry-mixed for 1 min. After adding the activator and water, the mixture was stirred for 2 min at low speed, followed by 2 min at high speed (with a 20 s interval between the two). After pouring into the mold, the mixture was vibrated 60 times. The mold was then covered with plastic wrap and placed in a curing chamber at (20 ± 2) °C and 95% relative humidity. After demolding 24 h later, the specimens were cured to the specified age. Subsequently, the test specimens were placed in a standard curing chamber at (20 ± 2) °C and 95% relative humidity for continued curing until the predetermined age, at which point, performance testing was conducted.

### 2.4. Test Methods

#### 2.4.1. Flowability Test

The fluidity of the geopolymer paste was assessed according to GB/T 8077-2023 [31]. The average result from three parallel test groups was taken as the fluidity value for each geopolymer sample.

#### 2.4.2. Setting Time Measurement

The testing method for the initial and final setting times of the geopolymer slurry is based on the standard (GB/T 1346-2024 [32]).

#### 2.4.3. Compressive Strength Test

The compressive strength test of the geopolymer net slurry was conducted in accordance with the standard (GB/T 17671-2021 [33]). Take the average of the measurement results from three parallel test groups as the compressive strength of the geopolymer sample. Cubic specimens measuring 40 mm × 40 mm × 40 mm were prepared and loaded at a rate of 2.4 (±0.2) KN/s.

The calculation formula is as follows:

$$R_{c}=\frac{F_{c}}{A}$$

where $F_{c}$ is the maximum load (N) the specimen can withstand at failure and A is the area of the compressed ${mm}^{2}$ region.

#### 2.4.4. Geopolymerization Heat

The geopolymerization heat and heat release rate of cementitious material paste were measured using an ICAL8000HPC isothermal calorimeter (Calmetrix, Nokomis, FL, USA). Freshly mixed paste was placed in a sample vial and inserted into the calorimeter maintained at 20 °C. Measurements were recorded continuously for 72 h [34].

### 2.5. Microstructural Analysis

#### 2.5.1. TGA

The pyrolysis behavior of the material was analyzed using a TGASTA4493C thermogravimetric analyzer (Netzsch, Selb, Bavaria, Germany) to quantify mass changes at different temperatures and elucidate the cementation reaction mechanism. During testing, samples were placed in a nitrogen atmosphere and heated from 30 to 1000 °C at 20 °C/min. The samples used in the test were geopolymer powders that had undergone 28 days of strength testing and were subsequently treated with anhydrous ethanol to terminate the hydration reaction.

#### 2.5.2. XRD

Phase composition was analyzed using a D8 Advance X-ray diffractometer (Bruker, Billerica, MA, USA). Scans were performed over a 2θ range of 5° to 80° at 5°/min. Test samples were geopolymer powders that had undergone strength testing after being soaked in anhydrous ethanol for 28 days.

#### 2.5.3. SEM and EDS

SEM analysis was performed using an Inspect F50 scanner (Thermo Fisher Scientific, Waltham, MA, USA) equipped with an SU822 energy dispersive spectrometer, operating at 5.0 kV with an SE2 detector (Carl Zeiss, Oberkochen, Germany). The surface morphology of the samples was observed and photographed. Subsequently, X-ray energy-dispersive spectroscopy (EDS, Thermo Fisher Scientific, Waltham, MA, USA) was employed to analyze the geopolymer samples, determining their elemental composition and relative abundances. Dry the geopolymer powder samples after the 28-day strength test. A suitable amount of the sample was affixed to a sample stage using double-sided tape, and a near-gold coating was sprayed onto it via an ion sputtering apparatus to enhance conductivity and improve the contrast of the scanning electron microscope signals.

## 3. Results and Discussion

### 3.1. Flowability

**Figure 4:**
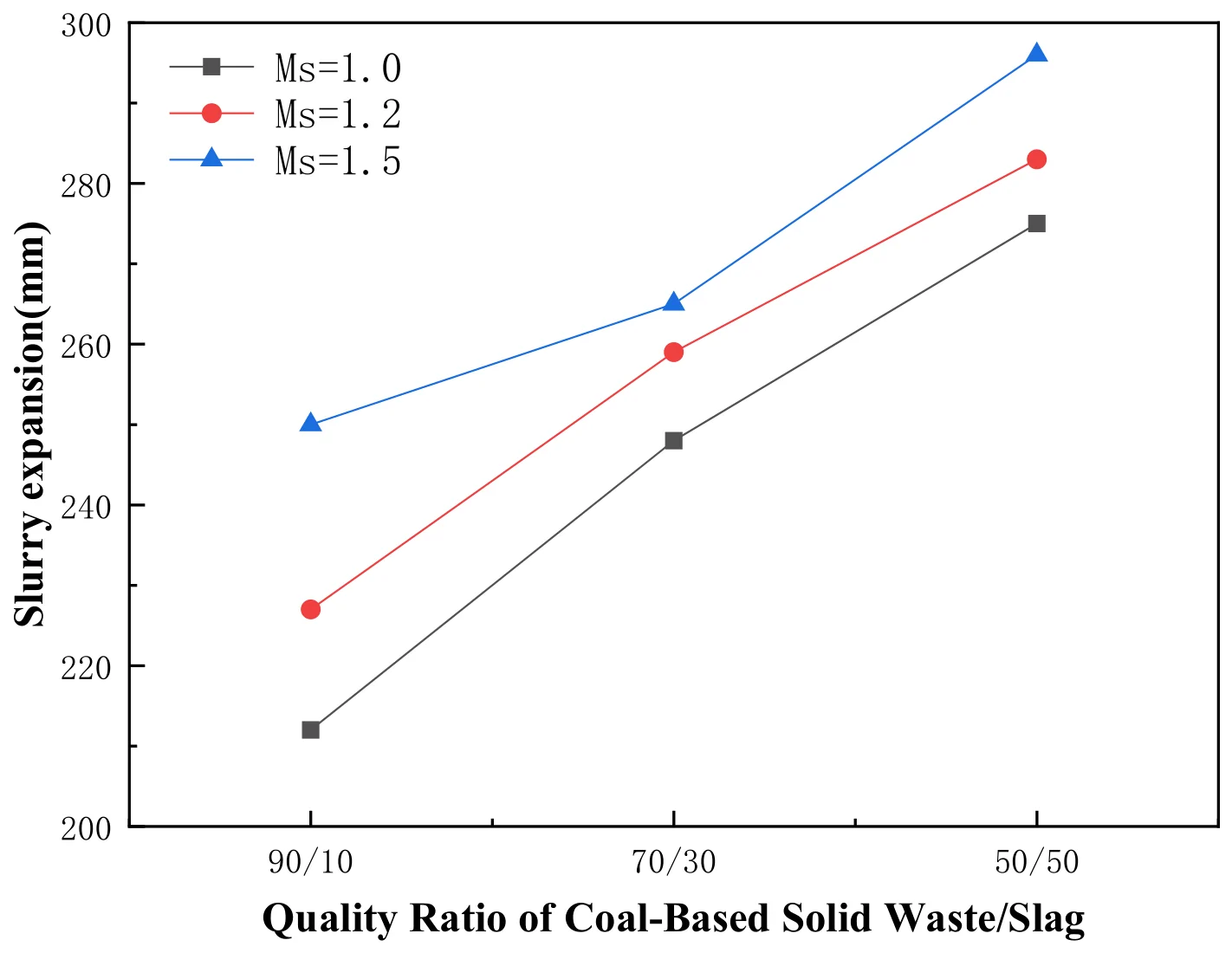
Effects of different factors on the expansion degree of geopolymer slurry.

Figure 4 illustrates the flowability variation patterns of geopolymer systems with different compositions. When the alkali activator remains constant, the flowability of geopolymer slurries increases significantly with reduced gangue content and increased slag content in coal-based solid waste. For example, in a system with a modulus of 1.5, the expansion of a 50% slag-containing sample reached 296 mm, representing an 18.4% increase compared to the 10% slag sample. At fixed coal-based solid waste/slag mass ratios, slurries’ flowability shows a slight upward trend with increasing alkali activator modulus, though the increase is relatively minor. When slag content reaches 50%, the expansion only increases by 7.6% when the modulus rises from 1.0 to 1.5.

Under controlled water-content conditions, the fluidity changes in geopolymer systems can be attributed to two key mechanisms. Firstly, as the coal gangue content increases, the C-A-S-H and N-A-S-H gels formed during early reactions interlink to create flocculated structures that encapsulate free water. This process simultaneously reduces the system’s free water content by enhancing water absorption, thereby decreasing expansion [35]. Secondly, the increased modulus of the alkali activator lowers the alkaline solution concentration in the system, thereby decreasing the slurry’s reaction rate. This results in higher water content in the mixture, thereby improving its fluidity [36].

### 3.2. Setting Time

**Figure 5:**
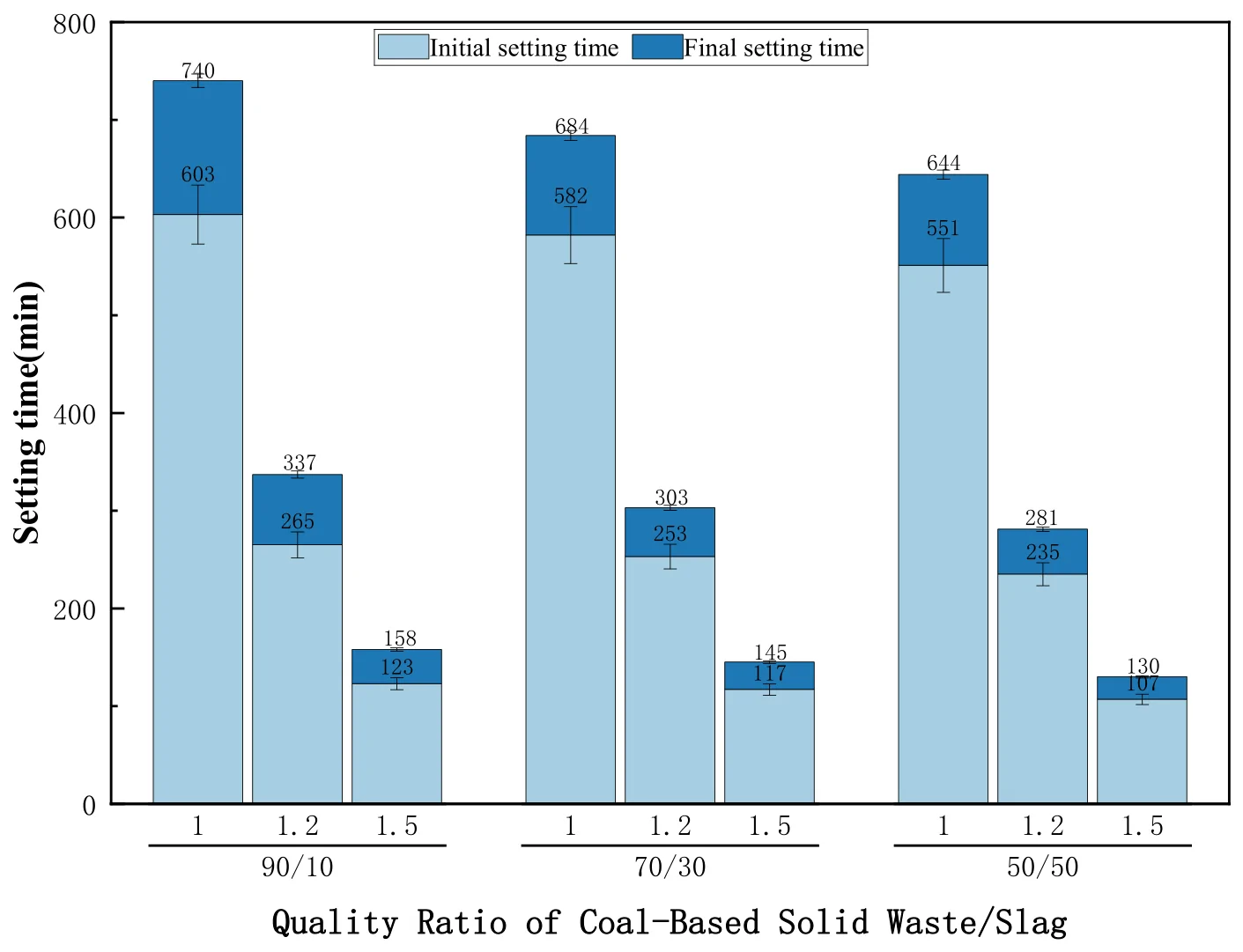
Effects of different factors on the setting time of geopolymer slurry.

Figure 5 demonstrates the effects of the coal-based solid waste/ground granulated blast furnace slag mass ratio and the alkali-activated agent modulus on the geopolymer setting time. This study reveals that increasing the ground granulated blast furnace slag content significantly shortens the geopolymer’s setting time: when the alkali-activated agent OH^−^SiO_4_^4−^AlO_4_^5−^ modulus is 1.2, the initial setting time decreases by 30 min as the slag content increases from 10% to 50%. This is attributed to the high reactivity of the slag, which rapidly hydrates under the alkali-activated agent and undergoes condensation-polymerization reactions with siloxane tetrahedra (SiO_4_) and aluminosilicate tetrahedra (Al_2_O_4_), accelerating gel formation [37]. Furthermore, under fixed mass ratios, increasing the alkali-activated agent modulus significantly enhances the setting rate. For instance, in samples with 50% slag content, the initial setting time reduces by 128 min when the modulus increases from 1.2 to 1.5. This phenomenon is closely related to the high-modulus activator’s ability to increase free Si content in the system, accelerate the dissolution of precursor active components, and promote gel formation [38].

The final setting time shows a consistent trend with the initial setting time, whereas the GGBS admixing ratio has a more significant effect on it. Taking the modulus 1.2 system as an example, when the slag content increases from 10% to 50%, the initial setting time shortens by 11.3%, while the final setting time decreases by 16.6%. This occurs because higher slag content triggers intense early-stage reactions in the geopolymer, where gel products rapidly fill pores and accelerate strength development, significantly shortening the interval between initial and final setting [39]. These results indicate that, when applying such geopolymer materials to stabilized recycled aggregates, strict control of the operation time from mixing to static pressure molding is essential to ensure the initial setting time is not exceeded, thereby guaranteeing molding quality.

### 3.3. Compressive Strength

**Figure 6:**
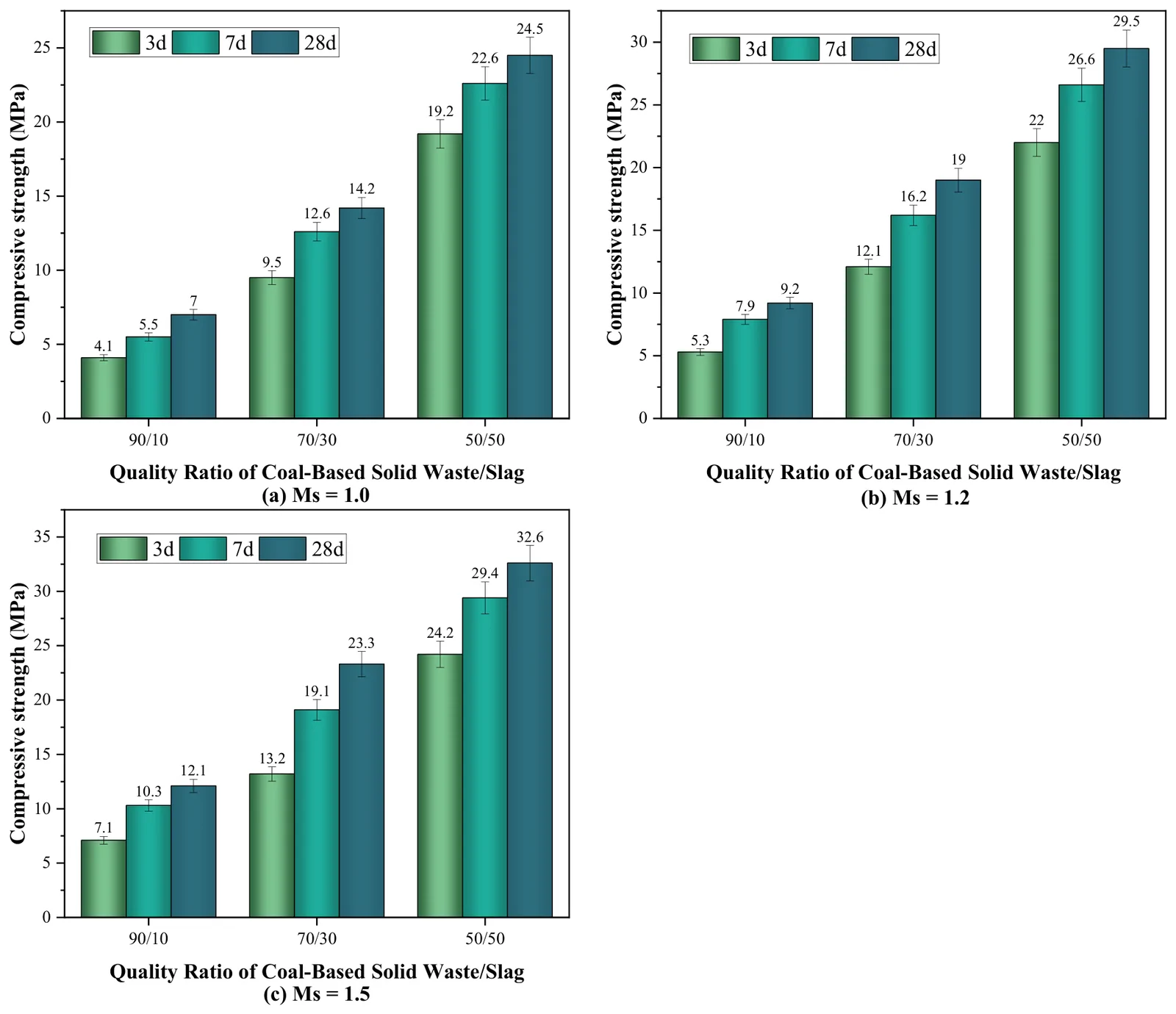
Effect of different factors on the compressive strength of geopolymer.

The compressive strength of the geopolymer slurry is illustrated in Figure 6. Results demonstrate that the compressive strength of geopolymer at various ages increases with increasing slag content. When the alkali activator modulus is 1.5 and slag content reaches 50%, the 28-day compressive strength of the geopolymer reaches 32.6 MPa, indicating its potential to replace cement-based binders. Higher slag content accelerates early-stage reactions by facilitating rapid dissociation and dissolution of Ca–O bonds under strong alkaline conditions. During the initial reaction phase, Ca^2+^ rapidly forms C-(A)-S-H gels [40]. For instance, at a modulus of 1.5, the 7-day strength of samples with 50% slag content increases by 185% compared to those with 10% slag content. However, the effect of coal gangue content shows the opposite trend: when the modulus is 1.2 and slag content decreases from 50% to 10%, the 7-day compressive strength of hardened slurry drops by 70%. This is attributed to the high crystallinity of medium-low temperature calcined coal gangue, which limits its reactivity in alkaline environments. The gangue primarily reacts with fly ash and slag to form a gel encapsulation layer, hindering further dissolution [41].

**Figure 7:**
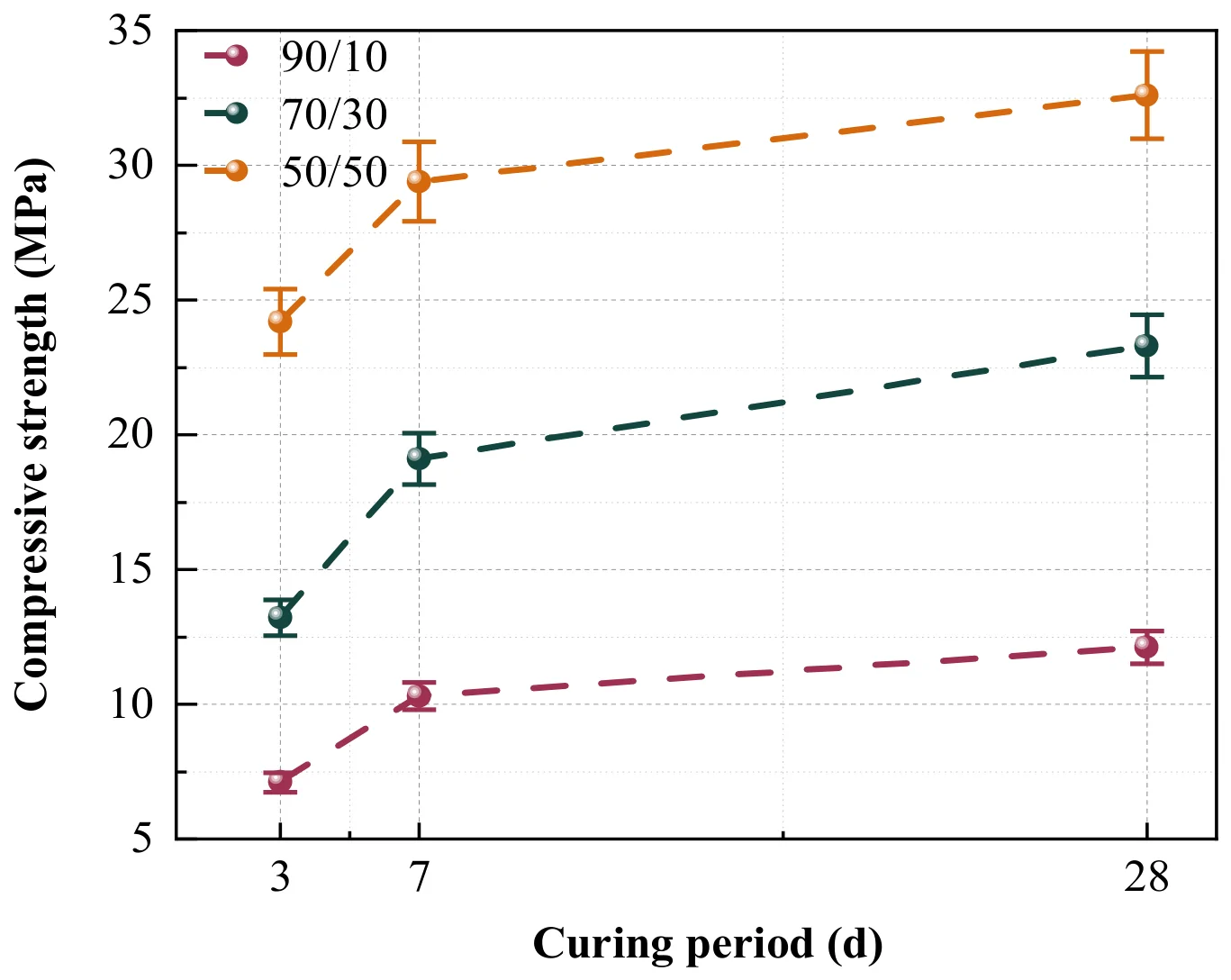
Changes in the compressive strength of geopolymers with Ms = 1.5 at different ages.

Figure 7 illustrates the compressive strength variation of geopolymer with Ms = 1.5 under different slag contents during curing. The data reveals that the strength growth primarily occurs within the first 7 days. When the alkali activator modulus is 1.5 and the slag content reaches 50%, the 7-day compressive strength of the slurry reaches 90% of its 28-day strength, demonstrating significant early strength. This phenomenon ${OH}^{-}{SiO_{4}}^{4-}{AlO_{4}}^{5-}$ stems from the rapid progress of geopolymerization reactions in the initial phase, where gel formation mainly occurs before day 7. As curing progresses, unreacted precursor materials like fly ash and coal gangue are further eroded, releasing active Si and Al elements that exist in specific structural forms. These elements ultimately form C-A-S-H gel products, explaining the slight strength enhancement observed at 28 days [37].

### 3.4. Microanalysis

#### 3.4.1. XRD Analysis

**Figure 8:**
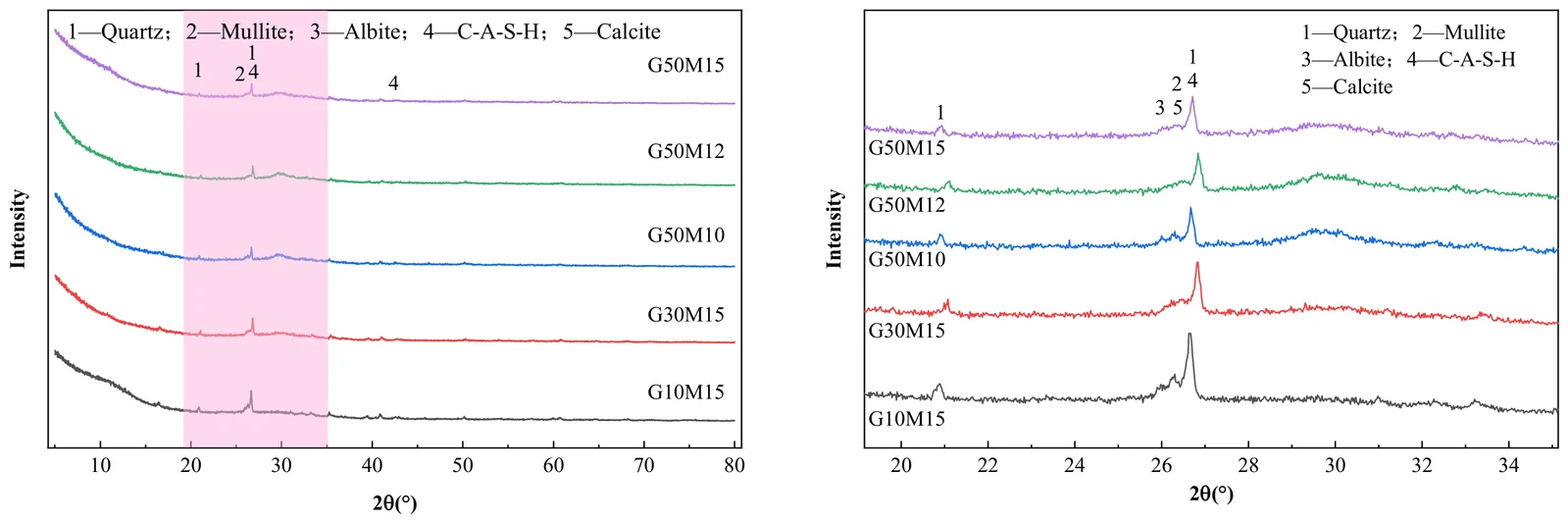
XRD test results of geopolymer.

XRD analysis was performed to explore how precursor composition and alkali activator modulus affect the phase evolution of the geopolymers. Figure 8 displays the diffraction patterns. A broad hump in the 20–40° 2θ range reflects the amorphous geopolymeric gel phase, the degree of which directly correlates with mechanical performance [42,43]. A comparison of specimens with different slag contents but the same modulus (e.g., G10M15 and G50M15) revealed that, as the slag content increased, the intensity of the diffraction peaks attributed to sodium feldspar (2θ ≈ 22°, 28°) significantly weakened or even disappeared. Since geopolymerization typically occurs at room temperature (≈20 °C), the energy barrier required for the low-temperature formation of crystalline albite is extremely high (requiring high temperatures or prolonged hydrothermal conditions), and therefore, the attenuation of these characteristic peaks does not imply that albite was consumed in large quantities during the reaction; rather, it is more likely due to the “dilution effect” of the high slag content on the precursors (fly ash and coal gangue)—that is, the total amount of unreacted residual crystals per unit mass of the sample decreases as the proportion of coal-based solid waste decreases. However, the peak shift (toward higher 2θ angles) of the diffuse “hump” in the 20–40° range of the XRD pattern provides direct evidence of the reaction extent. This shift indicates that an increased slag content promotes the formation of an amorphous C-(A)-S-H gel with higher polymerization and a larger Ca/Si ratio, rather than the formation of new crystalline phases [44]. The evolution pattern of this amorphous gel is in close agreement with the densification of the matrix and the increase in the Ca/Si ratio observed by SEM-EDS (see Table 4).

When the precursor material composition remains fixed (coal-based solid waste/ground granulated blast furnace slag = 50/50), the increase in the modulus of the alkali-activated agent does not promote the formation of sodium feldspar (as evidenced by the XRD spectra of G50M12 and G50M15). When the slag content is higher, the chemical reactions in the system are primarily dominated by the slag. Although the high-modulus activator increases the free silicon content, the silicon element preferentially combines with calcium to form C-(A)-S-H gel, inhibiting the formation of sodium feldspar [45]. Additionally, the low fly ash content and insufficient activity of coal gangue in the G50M15 sample limit the conditions for N-A-S-H gel formation, making it difficult to detect distinct characteristic peaks even when the modulus is increased.

The shift of amorphous characteristic peaks within the 20–40° range indicates that increased slag dosage shifts the diffraction peaks toward higher 2θ angles, demonstrating a significant increase in the polymerization degree of the sol-gel polymer. This phenomenon confirms that slag incorporation enhances system reactivity under strong alkaline conditions, thereby optimizing mechanical properties. The mineral composition changes in XRD spectra further confirm that CGP, FA, and GGBS collaboratively participate in the aggregation reaction, assisting in the formation of the gel phase.

#### 3.4.2. Analysis of Geopolymerization Heat

**Figure 9:**
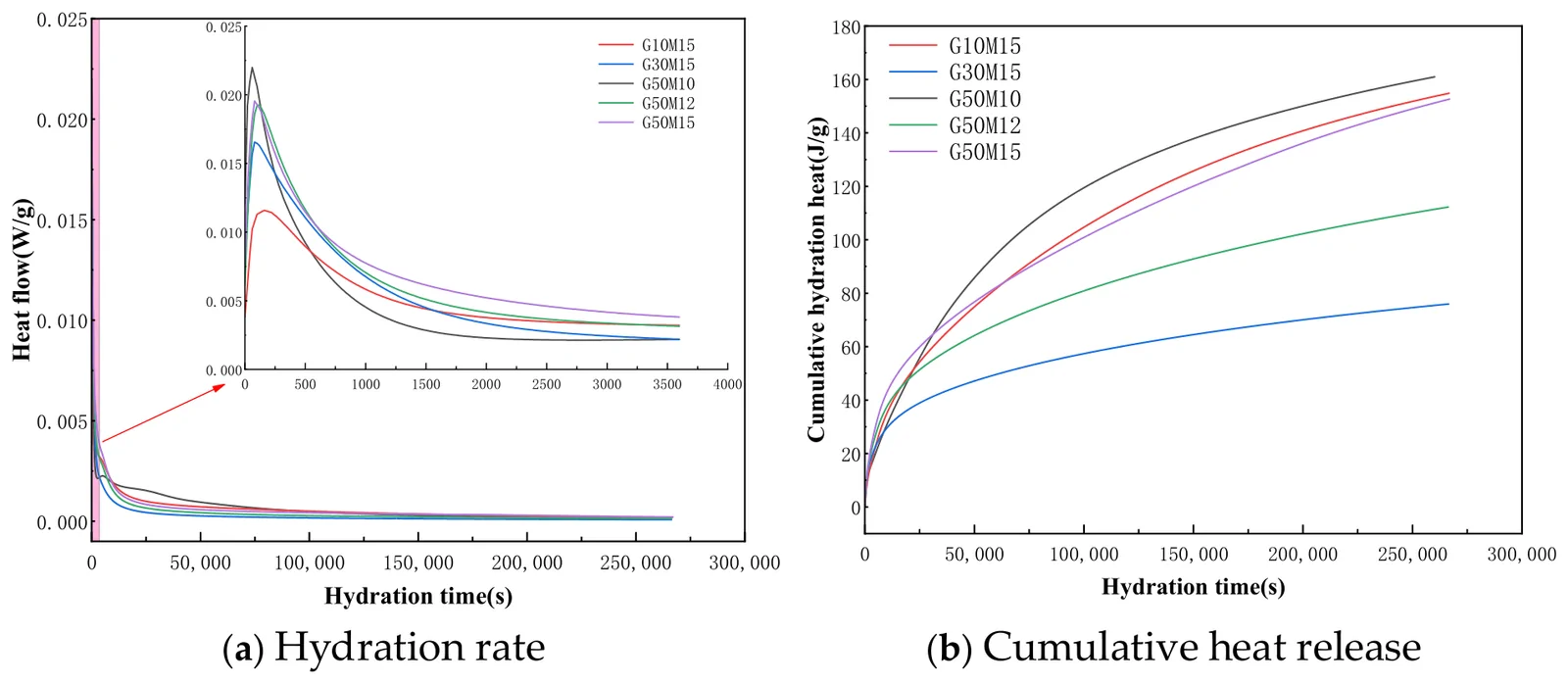
Hydration rate and cumulative geopolymerization heat curve of geopolymer.

Figure 9a reveals the thermal flux characteristics throughout the entire duration: all mixtures exhibit a “rapid rise–rapid decline–gradual decay” trend, corresponding to the typical alkali-activation kinetics phase of “initial rapid initiation → abrupt rate drop during induction → sustained slow progression in the later stage” [46]. Notably, G50M10 exhibits the lowest overall thermal flux, which aligns with the cumulative geopolymerization heat results and further confirms its slowest hydration reaction rate.

During the early hydration phase (0 s~3600 s), kinetic differences among different mineral powder ratios become more pronounced. G30M15 exhibits the highest peak heat flux at the earliest stage, demonstrating exceptionally strong early alkali-activation kinetics. G10M15 follows with a longer duration, exhibiting a “moderate and sustained rate” characteristic, indicating that the mineral powder content exerts a certain promotional effect on the hydration reaction. When the mineral powder content reaches 50%, the peak heat flux gradually increases as the accelerator modulus rises from 1.0 to 1.5, suggesting a dose-dependent effect of the accelerator modulus on hydration. Appropriate amounts of accelerators can stimulate hydration through the volcanic ash effect and micro-aggregate effect, while few mineral powder or low-activity accelerators may inhibit the reaction [47].

Figure 9b demonstrates that the cumulative geopolymerization heat increases monotonically with hydration time across all mixtures, confirming the ongoing hydration process and the continuous formation of hydration products. Notably, significant differences emerge between mixtures: when the accelerator modulus is 1.5, the cumulative geopolymerization heat rises as the slag content rises from 10% to 50%. This variation stems from changes in the composition of the cementitious material. The 50% slag mixture exhibits more complete hydration due to its higher proportion of rapid alkali-activated mineral phases, whereas the 10% mixture shows suppressed total heat release as the proportion of active hydration phases decreases [48].

#### 3.4.3. TG Analysis

**Figure 10:**
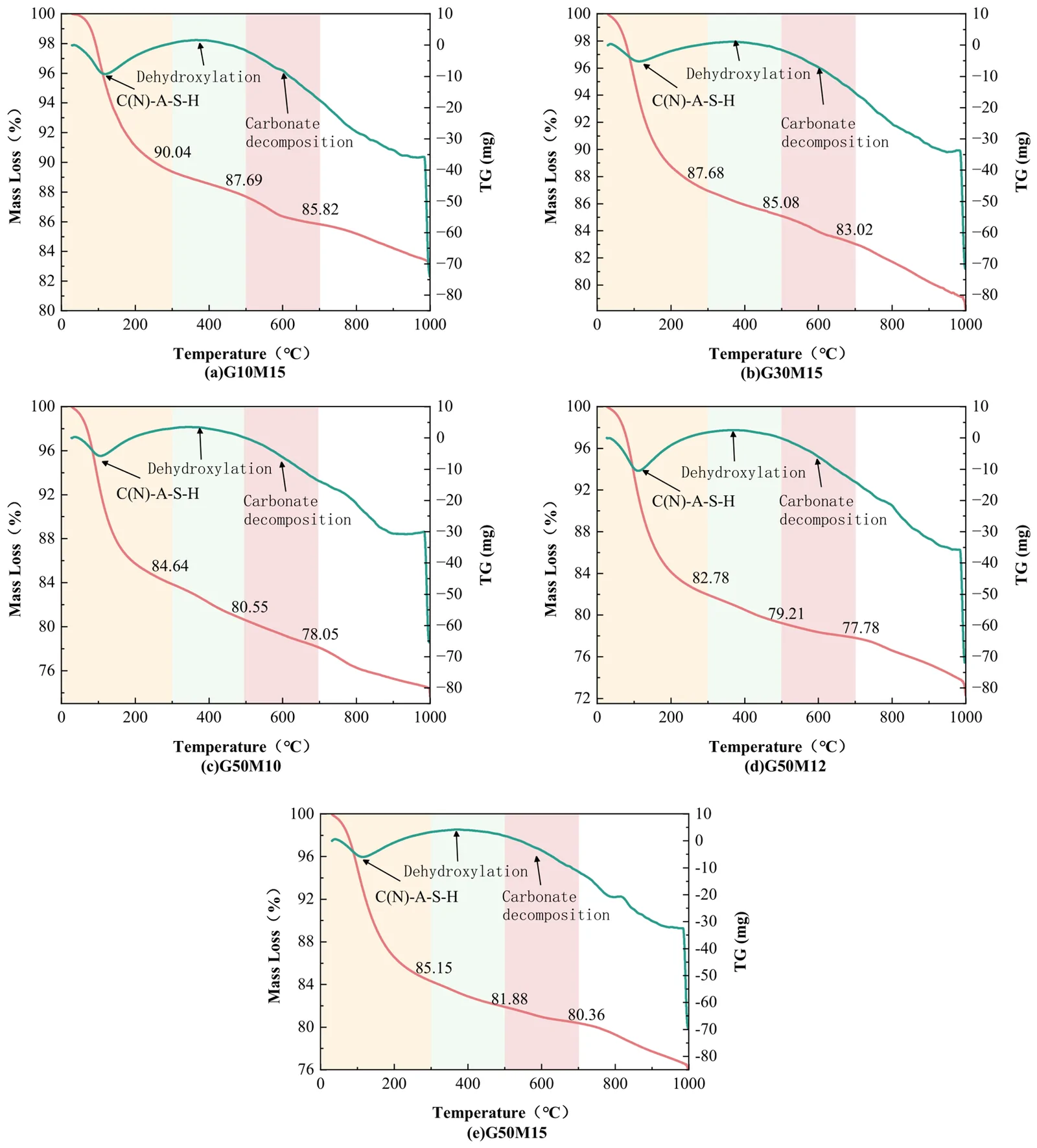
Mass loss and TG curve of geopolymer.

The thermogravimetric analysis in Figure 10 reveals three distinct mass loss phases in the TG/DTG curve of the geopolymer: below 250 °C, the C(N)-A-S-H gel-bound water evaporates; between 250 and 500 °C, dehydroxylation of hydrated minerals occurs; and from 500 to 700 °C, decomposition of carbonized products is observed [49]. The mass loss patterns in each phase exhibit strong correlation with the reaction kinetics of geopolymerization heat, demonstrating a clear coupling mechanism that elucidates the transition from reaction progression to product characterization.

The mass loss mechanism at the first stage (<250 °C) shows that coal gangue content significantly affects bound water content. When the modulus is 1.5 and slag content reaches 50%, the geopolymerization heat exhibits a “moderate peak followed by continuous decay” pattern, indicating that high slag activity promotes uniform and complete hydration. Notably, when the alkali-excitation modulus is 1.5, the mass loss rate of samples with 50% slag content reaches 14.85%, compared to 9.96% for those with 10% slag content. This confirms that higher coal gangue content hinders gel formation due to unreacted particles, reducing bound water content and causing free water to be encapsulated by flocculation structures [50]. These findings further indicate that fly ash content is a critical factor influencing substrate polymer fluidity. Increased slag content promotes greater free water participation in C-A-S-H gel formation, with mass loss rates increasing after heating-evaporation [51]. The effect of the alkali activator modulus shows a trend of first increasing and then decreasing: as the modulus increased from 1.0 to 1.2, the mass loss rate of the 50% slag sample rose from 15.36% to 17.22%; as the modulus increased from 1.2 to 1.5, the mass loss rate then decreased from 17.22% to 14.85%. This phenomenon occurs because a higher activation modulus enhances the replenishment of free silicon, thereby promoting gel formation; conversely, an excessively high activation modulus weakens the alkaline environment, thereby inhibiting the dissolution of precursors [52].

In the second phase (250–500 °C), the geopolymer hydration reaction is predominantly gel-forming, with the geopolymerization heat primarily arising from gel formation and minimal involvement of hydration-related mineral reactions. Lower mass loss observed in TG analysis during this stage further confirms the limited formation of hydration minerals in the system. The core of the reaction centers on the formation of C-A-S-H and N-A-S-H gels, as evidenced by XRD analysis showing no distinct hydration mineral peaks. This explains why geopolymer setting is primarily governed by gel formation rate rather than by hydration mineral transformation. Simultaneously, it demonstrates that the shortened setting time with increased slag content stems from rapid C-A-S-H gel formation, not from dehydroxylation processes. In the third phase (500–700 °C), the G50M15 sample with the optimal slag-to-gel ratio exhibits the lowest carbonation product decomposition rate. Its geopolymerization heat shows “moderate peak duration” (peaking at 1800 s and maintaining 0.3 mW/g after 3600 s), indicating uniform and thorough internal reaction processes. The dense C-A-S-H gel network formed through alkali-activated slag reaction lays the foundation for enhanced macro-mechanical properties. Thermal analysis results further corroborate that slag’s high reactivity initially dominates gel-phase formation, while coal gangue’s inert reactivity contributes less to mechanical properties during the early stages. The modulus of alkali-activated agents influences gel phase composition and content by regulating silicon–calcium coordination relationships [50,51].

#### 3.4.4. SEM and EDS Analysis

**Figure 11:**
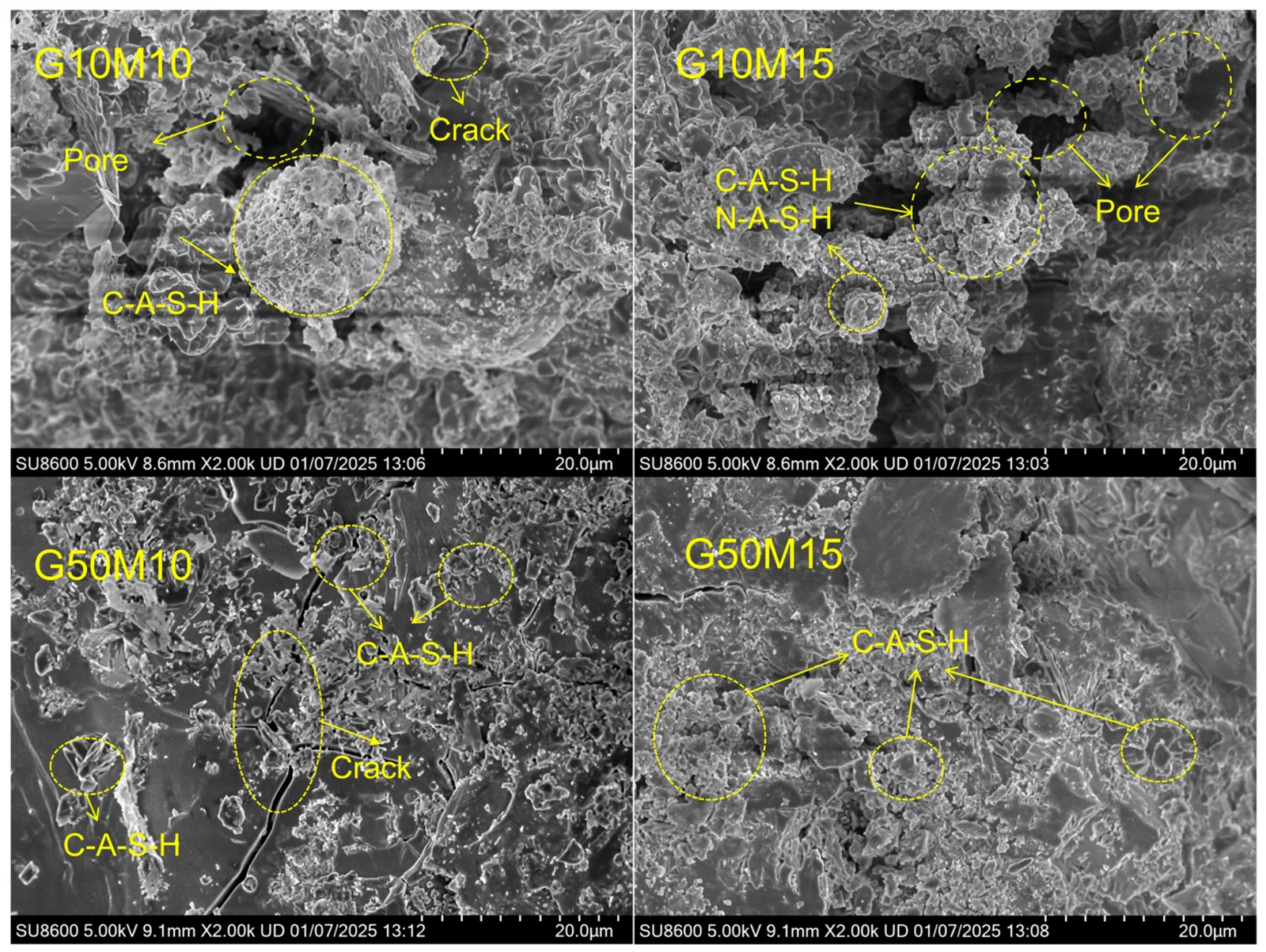
SEM images of fly ash–coal gangue–slag geopolymer.

Figure 11 illustrates the microstructural evolution of the fly ash–coal gangue–slag geopolymer. Comparative analysis of G10M15 and G50M15 samples reveals that increased coal gangue content results in a loose, porous matrix structure with abundant large-diameter pores. This phenomenon primarily occurs because higher coal gangue content reduces the availability of highly reactive precursors (fly ash and slag), leading to insufficient gel product formation ${OH}^{-}$ that fails to effectively fill matrix pores [53]. Additionally, coal gangue particles retain high-crystallinity minerals after medium-low temperature calcination, which remain insoluble at room temperature and thus cannot participate in the reaction. Unreacted coal gangue particles hinder gel network formation, leading to free water loss and further pore development [54,55]. The inadequate gel product formation may be one of the primary reasons for the insufficient strength observed in Section 3.3.

**Figure 12:**
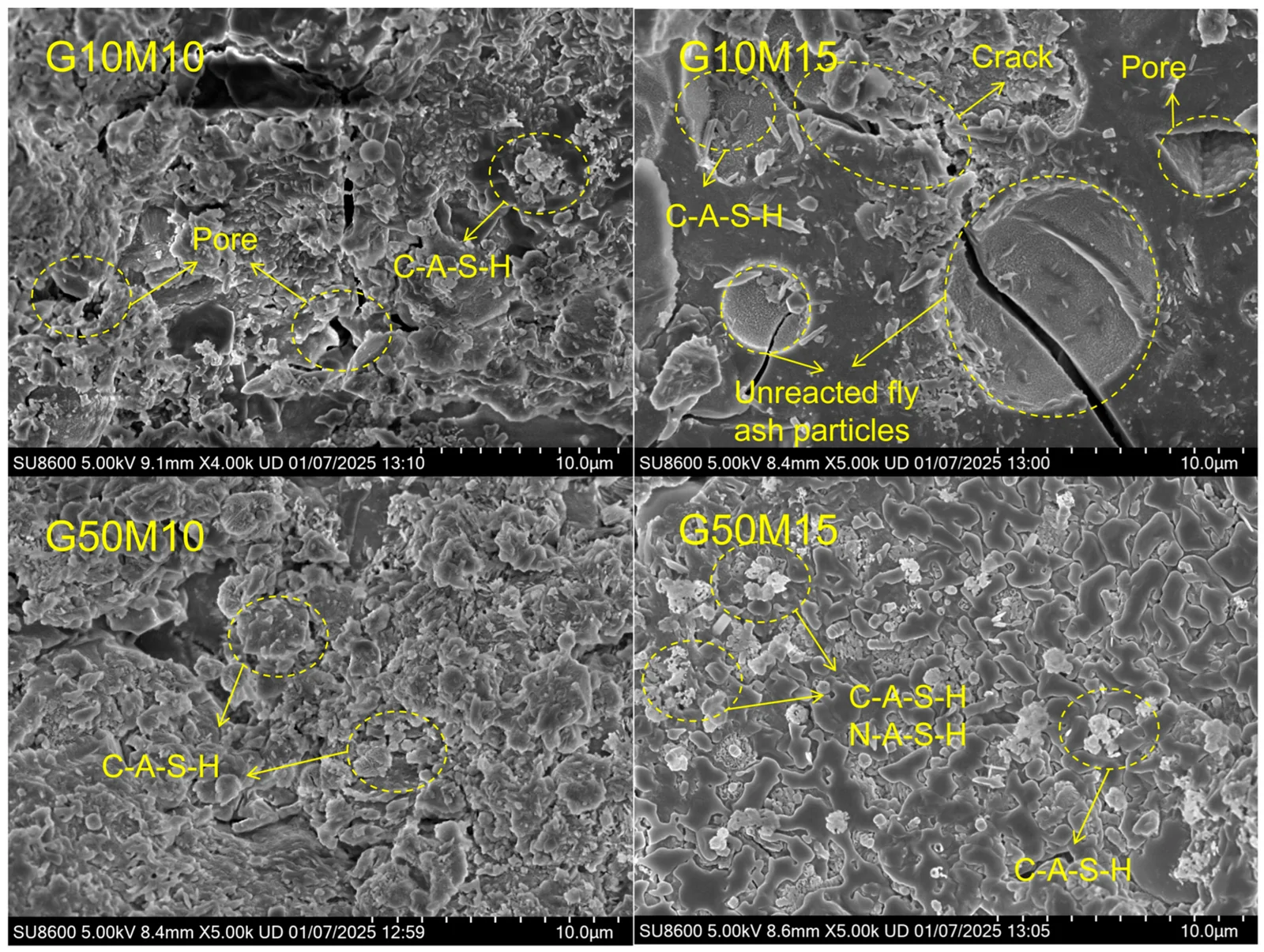
Partial magnification of the internal structure of the geopolymer-based composite materials of CGP, FA, and GGBS.

In contrast, systems with high slag content (e.g., G50M10, G50M15) exhibit a more compact microstructure. The high reactivity of the slag facilitates the thorough progress of geopolymerization and hydration reactions, generating substantial C-A-S-H gel phases. These gel phases effectively fill the matrix pores, while additional water exists as bound water within the gel network, reducing the loss of free water, which leads to a decrease in porosity [45]. Figure 12 further corroborates this finding, demonstrating that samples with higher coal-based solid waste content contain abundant unreacted particles. These particles form a dense physical protective layer, effectively inhibiting the erosion and penetration of OH^−^ into the matrix. This results in loose connections between gel products, which aligns with the mechanical property test results showing reduced strength due to increased coal gangue content [52].

EDS-determined atomic ratios (Na/Si, Ca/Si, Al/Si) for reaction products from selected samples are listed in Table 4. The data show a marked increase in the Ca/Si ratio of the C-A-S-H gel as the slag content increases from 10% to 50%. For 10% slag samples, Ca/Si ratios were only 0.25 and 0.27, whereas for 50% slag samples, they increased to 0.58–0.59, a growth exceeding 110%. This phenomenon indicates the core role of slag as a high-calcium precursor: its abundant CaO rapidly dissociates Ca^2+^ in alkaline environments and preferentially polymerizes with silicate to form C-A-S-H gel, significantly increasing the proportion of Ca^2+^ in the cementitious products [40]. Meanwhile, the Na/Si ratio exhibits an inverse trend: compared to 10% slag content, it does not increase with 50% slag content but slightly decreases at a modulus of 1.5. This suggests that, during the slag-dominated C-A-S-H gel formation process, Na^+^ primarily functions as a charge-balancing ion rather than a major component of the gel and its content is more significantly regulated by the activator modulus. The Al/Si ratio showed minimal variation across all formulations, indicating that aluminum dissolution and polymerization in the system are primarily determined by contributions from fly ash and coal gangue, while slag dosage exerts a relatively mild influence on the silico-aluminate coordination structure. Collectively, the atomic-ratio data from energy-dispersive spectroscopy (EDS) and SEM microstructural observations corroborate each other, establishing that slag dosage optimizes the system’s microstructure and macroscopic mechanical properties by regulating the Ca/Si ratio in the C-A-S-H gel.

## 4. Conclusions

This study successfully prepared geopolymer cementitious materials using fly ash, coal gangue, and slag as composite precursors, combined with composite silicate solutions as alkaline activators. The influences of material composition and activator modulus on mechanical properties and alkali-activation kinetics were systematically investigated. The primary conclusions are summarized as follows:

(1) As the slag content increases, the setting time of the slurry decreases, while its workability and compressive strength increase significantly; as the activator modulus increases, the slurry’s workability and compressive strength also show an upward trend. When the activator modulus is 1.5 and the slag content is 50%, the slurry’s 28-day compressive strength reaches its maximum value of 32.6 MPa.

(2) The heat of geological polymerization of the slurry exhibits a trend of “rapid rise—rapid decline—slow decay.” As the slag content increases, the cumulative geopolymerization heat gradually rises. The thermogravimetric analysis (TG) and differential thermal analysis (DTG) curves exhibit a three-stage mass loss profile, which is consistent with the evolution of the hydration reaction kinetics. The high reactivity of the slag accelerates the reaction process, but an increase in coal gangue content delays the formation of reaction products, thereby affecting the early performance of the system.

(3) Increasing the slag content promotes the formation of C-A-S-H gels with higher degrees of polymerization in geopolymer systems, resulting in a denser structure. Conversely, an excessive amount of coal gangue leads to a loose structure, and unreacted fly ash particles hinder the formation of the gel network.

Test results indicate that increasing the slag content and the modulus of the alkali activator can endow this geopolymer system with good workability and mechanical properties. The use of coal-based solid waste and industrial solid waste in geopolymers is feasible; it not only significantly reduces the consumption of natural resources but also substantially alleviates the environmental burden caused by the cement production process. However, current research is primarily limited to the mechanical properties and microstructural analysis of geopolymers, and studies on their long-term durability are still insufficient; future work will expand into these areas.

## Figures and Tables

**Table 1:** Main chemical composition of gelling materials.

Composition/wt.%	CaO	SiO_2_	Al_2_O_3_	Fe_2_O_3_	SO_3_	MgO	Na_2_O	K_2_O	LOI
CGP	0.20	50.78	46.53	0.58	0.03	0.18	0.06	0.14	0.12
FA	7.15	51.53	20.45	5.81	1.76	1.94	1.63	1.87	5.94
GGBS	43.65	30.12	9.87	1.25	2.94	6.03	1.29	0.76	2.09

**Table 2:** Composition of alkaline activators.

Alkaline Activator Modulus	NaOH	Sodium Silicate	Water
MS = 1.0	64.6	239.2	176.1
MS = 1.2	51.6	266.8	161.6
MS = 1.5	35.2	301.5	143.3

**Table 3:** Test mixtures of FA–CGP–GGBS geopolymers.

Order Number	Number	Slag Content	Alkali Excitation Modulus	CGP	FA	GGBS	Alkaline Activator
		(%)	(Ms)	(g)	(g)	(g)	(g)
1	G10M10	10	1.0	90	360	50	400
2	G30M10	30	1.0	70	280	150	400
3	G50M10	50	1.0	50	200	250	400
4	G10M12	10	1.2	90	360	50	400
5	G30M12	30	1.2	70	280	150	400
6	G50M12	50	1.2	50	200	250	400
7	G10M15	10	1.5	90	360	50	400
8	G30M15	30	1.5	70	280	150	400
9	G50M15	50	1.5	50	200	250	400

Note: The total weight of the precursor solids in each group is 500 (g). For every 500 g of slurry, 400 g of alkaline activator is required for activation. G10M15 indicates a GGBS content of 10% and an alkaline activator modulus of 1.5.

**Table 4:** Atomic ratios of geopolymers determined by energy dispersive spectroscopy.

Number	Na/Si	Ca/Si	Al/Si
G10M10	1.23	0.25	0.31
G10M15	0.84	0.27	0.33
G50M10	1.25	0.59	0.30
G50M15	0.82	0.58	0.27

## Data Availability

The original contributions presented in this study are included in the article. Further inquiries can be directed to the corresponding author.
